# Safety Evaluation of *Artocarpus altilis* as Pharmaceutical Agent in Wistar Rats

**DOI:** 10.1155/2014/980404

**Published:** 2014-04-02

**Authors:** Sudha Sairam, Asna Urooj

**Affiliations:** Department of Studies in Food Science and Nutrition, University of Mysore, Mysore 570006, India

## Abstract

This study was designed to elucidate the acute toxicity of *Artocarpus altilis* leaf and bark extracts. In acute toxicity study, no mortality or any toxic reaction was recorded in any group after 14 days of administering the extracts (2000 mg Kg^−1^ BW). The extracts (ALA, ABA, ALM, and ABM) did not cause any behavioural or physical changes in experimental rats. There was no significant (*P* ≤ 0.05) difference in the biochemical parameters analysed between the groups. Slight elevation in activities of AST and ALT in extract treated groups was observed, but this did not exert any deleterious effect on the normal metabolism which was supported by the histopathology of liver. Histopathological studies showed no remarkable changes after 14 days of oral administration of ALA, ABA, ALM, and ABM extracts. The study contributes to establishing the nontoxic quality parameters of *Artocarpus altilis* leaf and bark parts and the results suggest the safety of the extracts in therapeutic uses.

## 1. Introduction

India has an ancient heritage of traditional medicine. According to a survey by WHO, it is estimated that about 80% of people living in developing countries rely on traditional plant-based medicine for their basic health care needs [1, 2]. Natural products have numerous therapeutic approaches, contributed to understanding biochemical pathways, and are valuable tools in biological chemistry and molecular and cellular biology [3]. Plants used in traditional medicine contain wide range of ingredients that can be used to treat chronic as well as infectious diseases [4]. The potential medicinal value of plants lies in the bioactive compounds that produce a definite physiological action on the human body [5]. Bioactive compounds derived from medicinal plants can be useful but might have serious dose-related side effects. However, till now the dose-related toxicity of medicinal plants, particularly at the histological side, is not much known or explored [6]. Toxicity studies are conducted to assess the degree of toxicity of a component for humans, animals, or the environment, to investigate the mechanism of toxic chemicals, or to develop new or improved tests for specific types of chemically induced effects. The rationale for doing acute toxic study is to investigate the adverse effects that may occur on first exposure to a single dose of a substance and ensure its safe utilization. They also determine the maximum nonlethal dose and provide preliminary information relevant to single exposure or overdosage in humans. For pharmaceuticals, the results are used in combination with the efficacy of bioactives to decide whether the beneficial effects of the treatment would outweigh the risks of adverse side effects, if any, and to establish a safe dose for use in clinical trials [7]. These medicinal plants/metabolites then can be safely utilized as nutraceutical or can be incorporated into food formulation as functional ingredient.

*Artocarpus altilis* (Parkinson) Fosberg (Moraceae) is a tree of moderate size and is widely cultivated in tropics as staple crop, animal feed, and construction material. Its leaves have been used traditionally for the treatment of liver cirrhosis, hypertension, and diabetes [8, 9]. Pharmacological studies report the presence of flavonoids, [10, 11] triterpenoids [12], and prenylflavonoids [8] in* A. altilis* and some flavonoids have shown anti-inflammatory activities [13] and also inhibit 5-lipoxygenase of cultured mastocytoma cells [14]. The traditional usage of leaf to treat hypertension [15] and diabetes [16, 17] has been proven scientifically. Only few studies have been conducted to demonstrate the pharmacological or biological effects of* A. altilis*; however, there are no toxicological effects reported, and hence the present study is the first to be documented on the toxicity aspects.* A. altilis* is being explored for various biological activities such as antihyperglycemic activity [16, 17] in our laboratory and hence the present work was undertaken to study acute toxicological effect of various extracts of* A. altilis* leaf and bark parts.

## 2. Materials and Methods

### 2.1. Chemicals and Reagents

Alanine aminotransferase (ALT), aspartate aminotransferase (AST), alkaline phosphatase (ALP), total protein, albumin, urea, creatinine, total bilirubin, triglycerides, and total cholesterol assay kits were purchased from Aggappe Diagnostics, Ernakulam, India. Reduced glutathione (GSH) and 5,5-dithio(bis) nitrobenzoic acid (DTNB) were purchased from Sigma-Aldrich, Bangalore, India. All the chemicals and reagents used in the study were of analytical grade.

### 2.2. Collection and Preparation of Samples

The leaf and bark parts of* Artocarpus altilis* were collected from Mysore district of Karnataka, India, and subsequently identified by Dr. G. R. Shivamurthy, Department of Studies in Botany, University of Mysore, Mysore, India. The samples were thoroughly washed under running water to remove adhering dirt and other foreign particles from the surface, dried overnight (50°C), powdered, passed through 60 mesh sieve (BS), and stored in airtight container at 4°C till further use.

Various extracts of* A. altilis* leaf and bark used for* in vivo* biological experiments were studied for acute toxicological effect on animals according to OECD guidelines 420 [18]. The cold aqueous extracts of* A. altilis* leaf and bark were prepared by extracting powdered material with cold water (RT) in a mechanical shaker (24 h), filtered, and freeze dried in freeze drier (Thermo Modulyo D, Hong Kong). 80% of methanol extract was prepared by taking 15 g sample, extracted with 50 mL of 80% of methanol (methanol : water—8 : 2 ratio) in a mechanical shaker (6 hrs). The extracts were evaporated at 40°C under reduced pressure to dryness in a rotary evaporator (Superfit, India) and stored in air tight container at 4°C until further use. Sample codes are as follows: cold aqueous extracts; leaf—ALA, bark—ABA, 80% methanol extracts leaf—ALM, bark—ABM.

### 2.3. Experimental Animals

Adult Wistar strain albino rats weighing around 140–180 g were acclimatized for 14 days under standard conditions. The rats were housed in the polyacrylic cages, maintained at 25 ± 2°C, 45% to 60% RH, and 12 h photoperiod. During acclimatization period, the animals were observed for general conditions every day. Standard pellet diet (Amrut feeds, Pune, India) and water ad libitum were provided. The experimental protocol of toxicological studies was reviewed and approved by the Institutional Animal Ethical Committee (IAEC) for the purpose of control and supervision of experiments on animals (UOM/IAEC/29/2011).

### 2.4. Acute Toxicity Studies

The animals were grouped into 5 groups: group I—control; group II—ALA; group III—ALM; group IV—ABA; group V—ABM consisting of 6 animals each (3 males, 3 females) using randomized block design. According to OECD 420 guidelines, the animals were administered with 2000 mg kg^−1^ BW of extracts. The extracts were given in the form of suspensions, orally by gavage for 14 days. The animals were observed individually after the initiation of dose during first 30 min and at every half an hour interval for 6 hours and thereafter once in 24 hr for 14 days. Individual records were maintained to record physical or behavioural changes such as skin and fur, eyes and mucous membrane, respiratory, circulatory, autonomic and central nervous systems (ANS and CNS, resp.), somatomotor activity, behaviour pattern, and mortality. Observations were also made for presence of tremors, convulsions, salivation, diarrhoea, lethargy, sleep, and coma. At the end of the study period, animals were euthanized and decapitated.

### 2.5. Biochemical Estimations

Blood was collected and serum was separated after centrifuge at 2500 ×g for 20 min. Activities of alanine amino transferase (ALT), aspartate aminotransferase (AST), and alkaline phosphatase (ALP) were determined in serum along with estimation of total protein, albumin, urea, creatinine, total bilirubin, total cholesterol, and triglycerides (TGL) using respective standard kits. Glutathione (GSH) and thiobarbituric reactive oxygen species (TBARS) as markers of lipid peroxidation were determined by the methods of Ellman [19] and Ohkawa et al. [20], respectively, in serum, liver, and kidney homogenates.

### 2.6. Histopathological Procedures

Various organs like liver, kidney, heart, brain, and spleen were excised immediately, washed with phosphate buffered saline, and weighed. Small portions of liver were fixed in 10% formaldehyde, then dehydrated in graduate ethanol (50–100%), cleared in xylene, and embedded in paraffin. The sections (4-5 *μ*m) were stained with haematoxylin and eosin (H-E) dye and examined with photomicroscope (400x) for any histopathological changes.

### 2.7. Statistical Analysis

The values are expressed as mean ± SD. The data was subjected to one-way ANOVA followed by Tukey's multiple comparisons test for significant difference (*P* ≤ 0.05) using SPSS 16.0 software.

## 3. Results and Discussion

The yield of the different extracts was calculated and expressed as percentage (w/w). The leaf 80% MeOH extract gave maximum yield of 21.67% (w/w) followed by leaf aqueous extract (7.87%), bark 80% MeOH, and aqueous extracts (7.07% and 1.45%), respectively.

### 3.1. Acute Toxicity Studies

In the present acute toxicity study of different* A. altilis* leaf and bark extracts, the animals were treated with limit test dose of 2000 mg^-1 ^kg BW (OECD guidelines 420, 2001). The data on toxic symptoms and behavioural and other changes are presented as record sheet in Table 1. All the extracts did not show any toxic symptom during the study period. There were no significant changes in behaviour, ANS, or CNS and no mortality was observed in any of the animals. There was no significant (*P* ≥ 0.05) changes between weights of major organs in relation to their body weights (Table 2).

### 3.2. Biochemical Estimations

**Figure 1:**
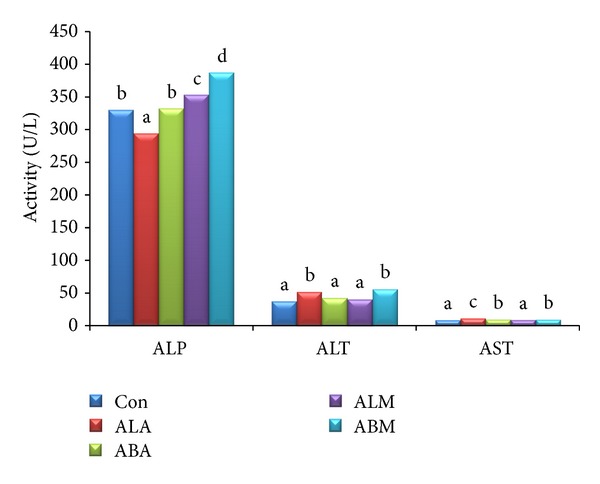
Serum hepatic enzymes levels of different groups (U L^−1^).

The activities of hepatic enzymes and selected biochemical parameters are represented in Figure 1 and Table 3, respectively. ALA and ABM showed significant (*P* ≤ 0.05) higher ALT activity. Although statistically there was significance (*P* ≤ 0.05) between the activities of ALP, ALT, and AST in experimental groups, it did not affect the normal metabolism or behavioural pattern. The data on serum total protein, albumin, creatinine, total bilirubin, and urea shown in Tables 3 and 4 contain the total protein and albumin contents in organs, namely, liver, kidney, and brain. There was no significant (*P* ≤ 0.05) difference observed in biochemical parameters between the groups except in the total protein and albumin contents of brain.

**Figure 2:**
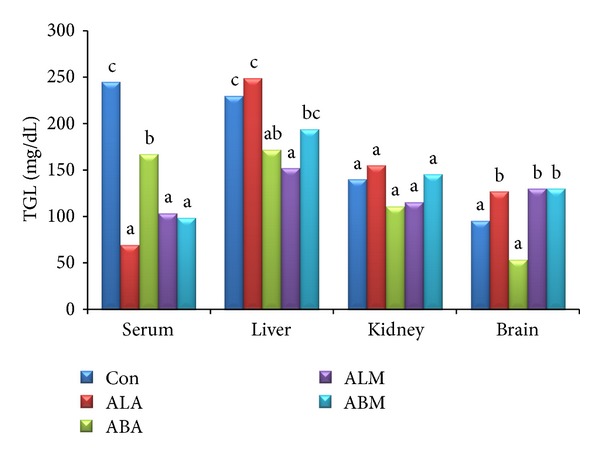
Triglyceride content of control and extract treated groups (mg dL^−1^).

**Figure 3:**
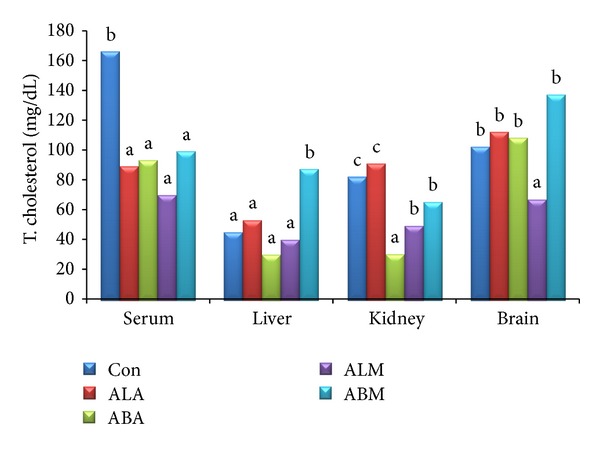
Total cholesterol content of control and extract treated groups (mg dL^−1^).

**Figure 4:**
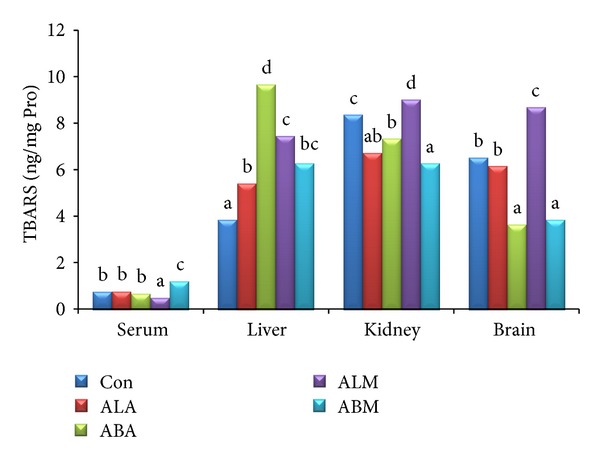
TBARS levels of control and extract treated groups (ng mg^−1^ Pro).

**Figure 5:**
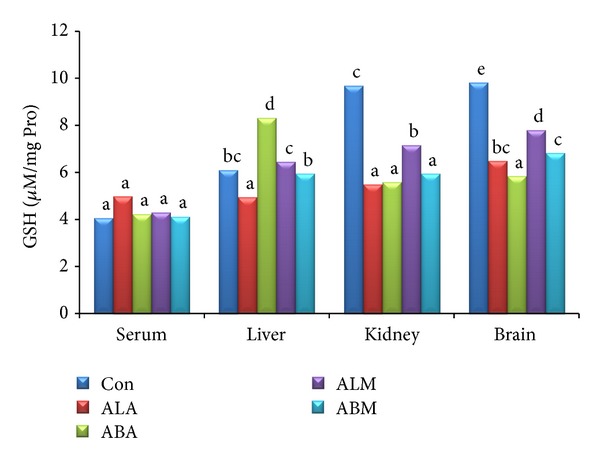
Glutathione levels of control and extract treated groups (*μ*M mg^−1^ Pro).

The triglyceride and total cholesterol contents in serum, liver, kidney, and brain of control and extract treated groups are shown in Figures 2 and 3, respectively. It was interesting to note that the total cholesterol levels were significantly (*P* ≤ 0.05) low in all the extract treated groups compared to control group. The TBARS (Figure 4) and glutathione (Figure 5) levels in serum along with liver, kidney, and brain homogenates were analysed in all the groups. The treatment with leaf and bark extracts did not show any adverse effects on cellular defence mechanisms against oxidative stress. The results suggested that leaf extracts performed better than the bark extracts.

### 3.3. Histopathological Procedures

**Figure 6:**
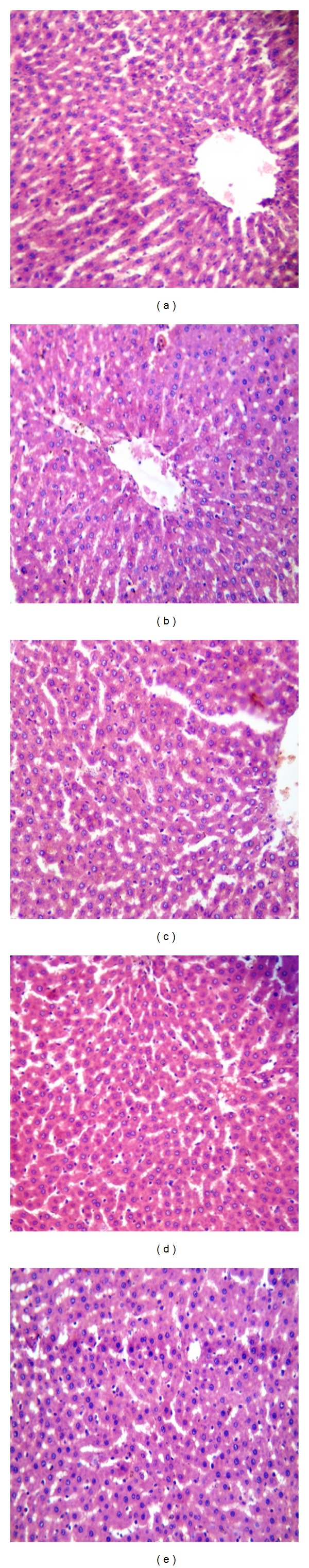
Histopathological changes in liver of control and extract treated groups. (a) Control; (b) ALA; (c) ABA; (d) ALM; (e) ABM.

The histological sections of the control and extract treated groups are represented in Figure 6. There were no detectable changes in cellular morphology of hepatocytes. The hepatic architecture was normal with well-defined central vein. No necrosis, steatosis, chronic inflammatory infiltration, or degenerative changes were observed in any of the extract treated animals. The biochemical and histopathology results of leaf and bark extracts are comparable, and the reason for these similarities may be that the samples are from the same plant. The preliminary investigation on the presence of phytochemicals in the extracts of* A. altilis* leaf and bark parts revealed that maximum phytochemical constituents were present in methanol extracts of both AL and AB. Terpenoids and triterpenoids were present in all solvent extracts of AL and AB. Saponins and tannins were present in aqueous extracts, while steroids were present in petroleum ether, benzene, and methanol extracts of AL and AB [21].

Medicinal/herbal plants and their preparations are being used from thousands of years in all types of traditional medicinal practices; one of the reasons is due to their nontoxic effect. They are rich sources of numerous bioactive components which can prevent, treat, and help in the management of several disease/disorders and till date only few plants have been explored for their potential pharmacological activities. Although the biological effects and bioactive components are identified, there is no scientific documentation of their toxicological effects.

Synthetic drugs usually consist of a single chemical, while medicinal plants have complex mixture of 400 or more chemicals. The side effects of a single compound or bioactive can be evaluated but in the crude plant extract it is very difficult to understand the complex interactions and synergies taking place between hundreds of plant metabolites [22]. These medicinal herbs contain numerous complex compounds, often mucilages, tannins, polysaccharides, and so forth which may modulate or modify the effects of bioactive components. Studies have also shown that the biological effects exerted by extracts of whole plants cannot be mimicked by administering isolated purified constituents of the plant [23]. Hence, it is very essential to assert the beneficial as well as the toxic effects of these plant derivatives before recommending and used as supplement or nutraceutical for any disease condition.

The description for any toxicity varies for foods and medicinal plant derivatives. Commonly consumed foods contain constituents that could be allergic or considered as toxic, to list a few, alpha gliadin produced by wheat, rye, and oats, cyanogenic glycosides present in many fruit seeds, lectins in pulses mainly in soya and red kidney beans, alkaloids present in Solanaceae family, thiocyanates of the Brassica vegetables, and so on. Despite the presence of these constituents, all the above foods are regarded as safe throughout the world; the main reason is the amounts or levels of allergic/toxic components present. Most components if taken in excess might reverse the affirmative effect. Similarly, water and oxygen which are essential for life can kill in excessive amounts, so the quantity is often an important consideration [24, 25].

The results confirm the safety of aqueous and 80% MeOH extracts of* A. altilis* leaf and bark parts, as it did not exert any allergic symptoms in experimental animals and also by observing physical and behavioural changes after administration of the sample, which is one of the simple ways to assert the toxic effects. The liver is the vital organ, involved in the maintenance of metabolic function and detoxification of drugs. If the normal metabolic function is hampered due to hepatic damage, there will be elevation in serum levels of hepatic health markers like ALT, AST, ALP, and bilirubin [26]. The hepatic enzyme activities and the biochemical parameters analysed proved the nontoxicity of the sample extracts. Although the activities of AST, ALP, and total bilirubin content differed significantly (*P* ≤ 0.05) in the groups, they did not exert any remarkable changes on the normal metabolic processes. Marginal elevation in the activities of AST and ALP extract treated groups may not be an indication of hepatic damage, which can be confirmed by other biochemical parameters and histopathology of liver.

## 4. Conclusion

*A. altilis* is used in traditional medical systems, time-tested, and used to treat various ailments till date. These traditions have successfully set an example of natural resource use in curing many complex diseases. From the results of this acute toxic study, it is inferred that* A. altilis* leaf and bark extracts are safe to be utilized as functional ingredient or as nutraceutical.

## Figures and Tables

**Table 1:** Acute oral toxicity record sheet for control and extract treated animals (*n* = 6).

				Additional observations																
Drug P.O	Toxicity		Time of death	ANS/CNS									Behavioural observations							
	onset	Stop		Skin and fur	Eye lacri	Sali	Diah	Resp	Leth	Sleep	Con	Coma	Ste	Tre	Cat	Geo	Hal	Retr	Stu	Exe
CON	nil	Nil	0	×	×	×	×	×	×	×	×	×	×	×	×	×	×	×	×	×
ALA	nil	Nil	0	×	×	×	×	×	×	×	×	×	×	×	×	×	×	×	×	×
ABA	nil	Nil	0	×	×	×	×	×	×	×	×	×	×	×	×	×	×	×	×	×
ALM	nil	Nil	0	×	×	×	×	×	×	×	×	×	×	×	×	×	×	×	×	×
ABM	nil	Nil	0	×	×	×	×	×	×	×	×	×	×	×	×	×	×	×	×	×

Con: control; ALA: leaf aqueous extract; ALM: leaf 80% MEOH extract; ABA: bark aqueous extract; ABM: bark 80% MEOH extract.

Lacri: lacrimination, Sali: salivation, Diah: diarrhoea, Resp: respiratory distress, Leth: lethargy, Con: convulsions, Ste: stereotypy, Tre: tremors, Cat: catalepsy, Geo: effect on positive geotropism, Ret: retropulsion, Stu: stupor, Exe: excitement, ×: absence of symptom, *✓*: presence of symptom.

**Table 2:** Relative body (BW) and organ weights of control and experimental groups (g) (values in parenthesis indicate organ-BW ratio) (*n* = 6).

Group	Initial BW	Final BW	Liver	Kidney	Heart	Brain	Spleen
Con	161^a^ ± 9.67	152^a^ ± 7.63	4.29^a^ ± 0.53 (2.28)	1.05^a^ ± 0.27 (0.69)	0.45^a^ ± 0.15 (0.29)	1.42^a^ ± 0.95 (0.93)	0.32^a^ ± 0.01 (0.21)

ALA	183^a^ ± 5.83	168^a^ ± 2.01	5.19^a^ ± 0.31 (3.08)	1.29^a^ ± 1.38 (0.76)	0.62^a^ ± 0.23 (0.36)	1.56^a^ ± 0.80 (0.92)	0.35^a^ ± 0.01 (0.20)

ALM	161^a^ ± 4.31	150^a^ ± 8.43	4.46^a^ ± 0.75 (2.97)	1.16^a^ ± 0.25 (0.77)	0.60^a^ ± 0.42 (0.40)	1.57^a^ ± 0.77 (1.04)	0.34^a^ ± 0.01 (0.22)

ABA	172^a^ ± 6.66	155^a^ ± 5.28	4.11^a^ ± 0.77 (2.65)	1.16^a^ ± 0.37 (0.78)	0.61^a^ ± 0.64 (0.39)	1.50^a^ ± 0.97 (0.96)	0.28^a^ ± 0.03 (0.18)

ABM	172^a^ ± 5.52	184^b^ ± 5.96	5.52^a^ ± 0.87 (3.00)	1.42^a^ ± 0.31 (0.77)	0.62^a^ ± 0.28 (0.33)	1.47^a^ ± 1.02 (0.79)	0.36^a^ ± 0.02 (0.19)

ALA: leaf aqueous extract, ALM: leaf 80% MeOH, ABA: bark aqueous extract, ABM: bark 80% MeOH extract.

Mean values carrying different superscripts a, b, c … in columns differ significantly (*P* ≤ 0.05).

**Table 3:** Changes in biochemical parameters in serum of control and experimental groups.

Group	T. pro (g dL^−1^)	Albumin (g dL^−1^)	Creatinine (mg dL^−1^)	T. bilirubin (mg dL^−1^)	Urea (mg dL^−1^)
Con	4.38^a^ ± 1.28	3.94^b^ ± 0.28	1.20^b^ ± 0.02	0.43^b^ ± 0.02	74^a^ ± 0.63
ALA	4.04^a^ ± 1.15	3.01^b^ ± 0.42	0.74^b^ ± 0.02	0.30^b^ ± 0.01	58^a^ ± 0.37
ABA	4.86^a^ ± 0.59	3.27^b^ ± 0.35	0.44^a^ ± 0.01	0.12^a^ ± 0.03	51^a^ ± 0.91
ALM	3.97^a^ ± 0.66	2.96^ab^ ± 0.75	0.60^b^ ± 0.15	0.41^b^ ± 0.01	63^a^ ± 0.84
ABM	4.17^a^ ± 0.98	2.03^a^ ± 0.32	0.60^b^ ± 0.18	0.61^c^ ± 0.03	72^a^ ± 0.65

ALA: leaf aqueous extract, ALM: leaf 80% MeOH, ABA: bark aqueous extract, ABM: bark 80% MeOH extract.

Mean values carrying different superscripts a, b, c … in columns differ significantly (*P* ≤ 0.05).

**Table 4:** Total protein and albumin contents in liver, kidney, and brain (g dL^−1^).

Groups	TP			Albumin		
	Liver	Kidney	Brain	Liver	Kidney	Brain
Con	1.51^b^ ± 0.82	0.44^a^ ± 0.12	0.73 ± 0.08	0.45 ± 0.02	0.45 ± 0.02	0.63 ± 0.03
ALA	1.49^b^ ± 0.76	0.76^a^ ± 0.29	1.15 ± 0.15	0.24 ± 0.05	0.64 ± 0.15	0.77 ± 0.08
ABA	0.71^a^ ± 0.25	0.53^a^ ± 0.10	0.98 ± 0.17	0.55 ± 0.07	0.5 ± 0.02	0.87 ± 0.06
ALM	1.15^ab^ ± 0.40	0.47^a^ ± 0.37	0.51 ± 0.08	0.66 ± 0.02	0.37 ± 0.05	0.31 ± 0.02
ABM	1.44^b^ ± 0.42	0.59^a^ ± 0.12	1.54 ± 0.21	0.83 ± 0.11	0.42 ± 0.05	1.26 ± 0.05

ALA: leaf aqueous extract, ALM: leaf 80% MeOH, ABA: bark aqueous extract, ABM: bark 80% MeOH extract.

Mean values carrying different superscripts a, b, c … in columns differ significantly (*P* ≤ 0.05).
